# Case Report: A *FBN1* frameshift-and-nonsense mutation and aortic dissection in Marfan syndrome

**DOI:** 10.3389/fcvm.2025.1533138

**Published:** 2025-04-23

**Authors:** Chao Su, Linjun Zeng, Haocheng Lu, Zanxin Wang, Minxin Wei

**Affiliations:** ^1^ Division of Cardiovascular Surgery, Cardiac and Vascular Center, The University of Hong Kong-Shenzhen Hospital, Shenzhen, China; ^2^ Department of Pharmacology, Joint Laboratory of Guangdong-Hong Kong Universities for Vascular Homeostasis and Diseases, School of Medicine, Southern University of Science and Technology, Shenzhen, Guangdong, China

**Keywords:** Marfan syndrome, fibrillin-1, mutation, aortic dissection, connective tissue disorder

## Abstract

**Background:**

Marfan syndrome (MFS) is an autosomal dominant connective tissue disorder primarily affecting the cardiovascular, ocular, and skeletal systems. Cardiovascular complications are the leading cause of mortality in MFS. Mutations in the *FBN1* gene, which encodes fibrillin-1, a critical extracellular matrix protein, are the predominant cause of the disorder.

**Case presentation:**

On March 11, 2024, we diagnosed a 30-year-old female proband with MFS based on the revised Ghent criteria, presenting with aortic root aneurysm, aortic dissection, multiple skeletal abnormalities, and a family history of MFS. Whole-exome sequencing followed by Sanger sequencing confirmation identified a novel inherited insertion mutation (c.4991dupA) in exon 40 of the *FBN1* gene. We performed valve-sparing aortic root replacement (David Procedure) and total aortic arch replacement using a tetrafurcated graft, along with the implantation of a specially designed frozen elephant trunk in the descending aorta (Sun's Procedure). Postoperatively, the patient underwent biweekly clinical follow-ups for three months. No treatment-related adverse events were reported during the monitoring period.

**Conclusion:**

The diagnosis of MFS requires an integrated approach, combining clinical manifestations, imaging studies, and genetic analysis. This novel mutation is associated with severe skeletal manifestations and life-threatening cardiovascular abnormalities, underscoring its clinical relevance. Its association with aggressive phenotypes enhances genotype-phenotype correlations. Importantly, these findings highlight the imperative need for early intervention in high-risk individuals by bridging genetic discovery to clinical practice.

## 1 Introduction

Marfan syndrome (MFS) is an inherited connective tissue disorder characterized by key features such as tall stature with arachnodactyly, ectopia lentis, and thoracic aortic aneurysm and dissection with an incidence of 1–2 per 10,000 (1). MFS can be life-threatening due to cardiac complications, particularly aortic aneurysm rupture. Early diagnosis is, therefore, crucial to enable timely prevention and appropriate treatment, significantly reducing the risk of severe outcomes.

Mutations in *FBN1* have been identified as the primary etiological factor (2). These mutations are distributed throughout the entire gene (3). As of March 2025, the Universal Mutation Database (UMD, http://www.umd.be/FBN1/) has documented 3,077 *FBN1* variants, while the ClinVar database (https://www.ncbi.nlm.nih.gov/clinvar/) includes 3,515 pathogenic or likely pathogenic variants (4). However, the genotype-phenotype correlations in MFS remain incompletely understood, necessitating ongoing research to elucidate the functional consequences of specific mutations.

The *FBN1* gene, spanning 230 kilobases (kb) on chromosome 15q21.1, consists of 65 coding exons (5). It encodes fibrillin-1, a multidomain protein composed of 2,871 amino acids, characterized by a highly repetitive structure (6). Fibrillin-1 contains 47 epidermal growth factor-like (EGF) domains, including 43 calcium-binding EGF (cbEGF) domains and 7 transforming growth factor-beta1 binding protein-like (TGFBP) domains (6). As a critical structural glycoprotein of the extracellular matrix, fibrillin-1 plays a vital role in supporting connective tissues, particularly in the arteries, the perichondrium, and the ocular tissues (7).

The identification of novel mutations in *FBN1* is crucial for improving our understanding of MFS pathogenesis, enhancing diagnostic accuracy, and developing targeted therapeutic strategies. Currently, challenges in MFS management include predicting disease progression and determining the optimal timing for preventive interventions, particularly regarding cardiovascular complications. Expanding our knowledge of genotype-phenotype correlations could potentially address these challenges by enabling more personalized risk stratification and treatment approaches.

## 2 Case presentation

**Figure 1:**
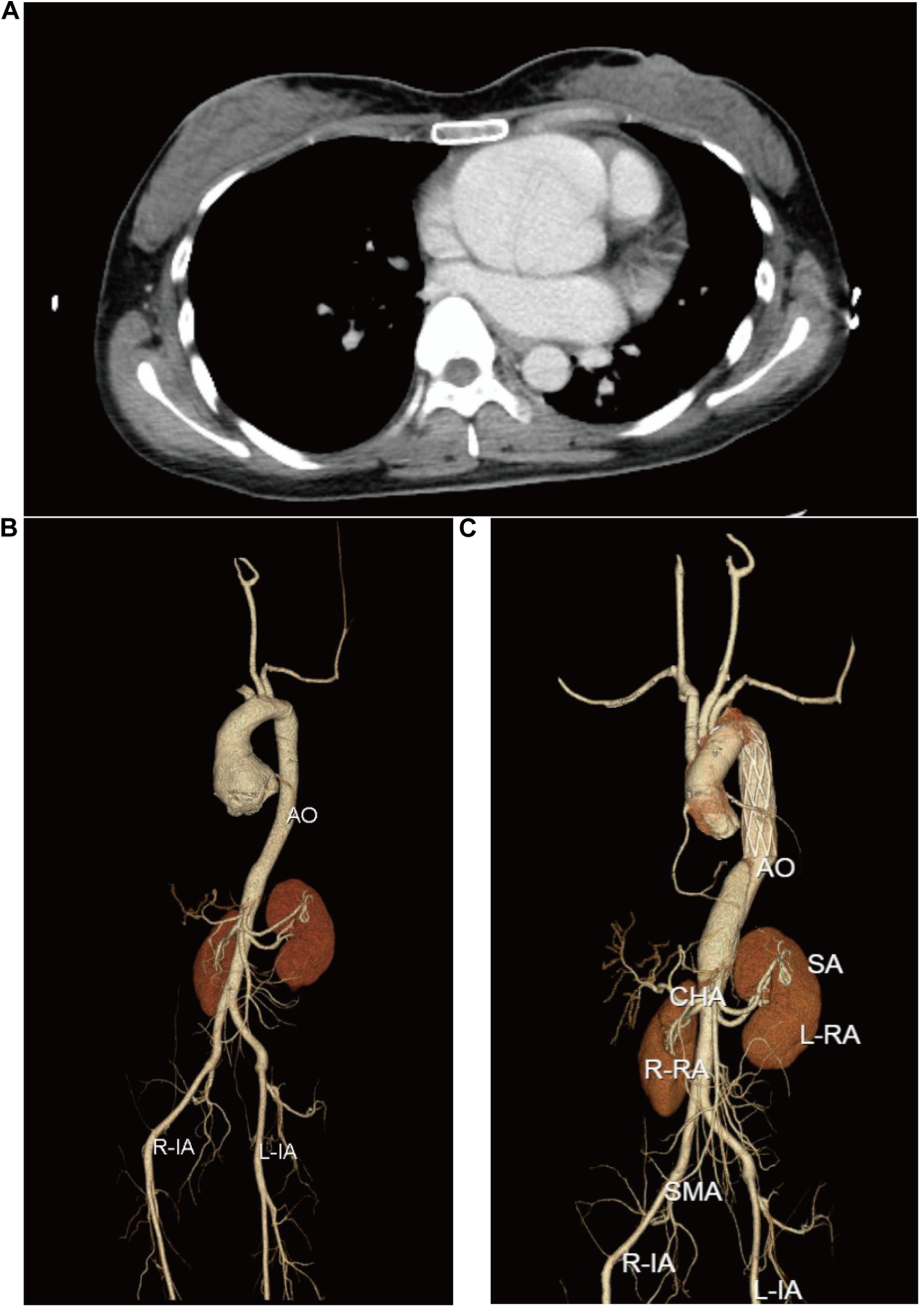
Cardiovascular disorders nin the proband. **(A)** CT image of the aortic root. **(B)** and **(C)** depict the multiplanar reconstructions of the aorta before and after David and Sun's procedure, respectively.

**Figure 2:**
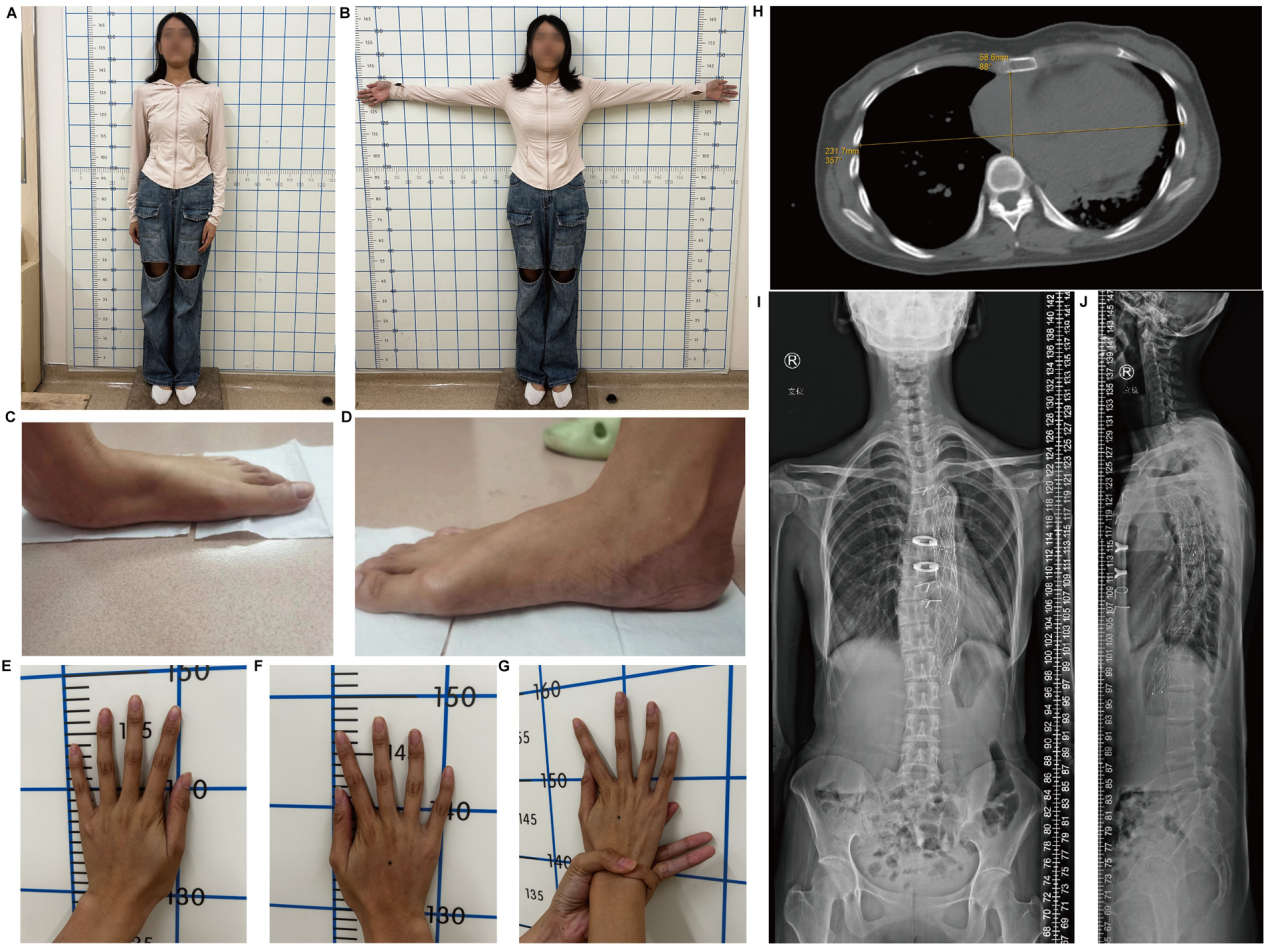
Skeletal abnormalities in the proband. **(A)** The height and **(B)** arm span of proband. **(C)** Left foot and **(D)** Right foot demonstrating pes planus. **(E)** Left hand and **(F)** right hand show arachnodactyly. **(G)** Positive wrist sign. **(H)** Chest x-ray image illustrating pectus excavatum. **(I)** Anteroposterior and **(J)** lateral x-ray film of the whole spine of the proband.

On March 11, 2024, a 30-year-old female proband was admitted to the hospital because of type-A aortic dissection. Imaging revealed significant cardiovascular abnormalities in the proband. Computed tomography (CT) scan demonstrated an aortic root aneurysm with a diameter of 65 mm (Z-score = 13.91) and aortic dissection (Figure 1A–C). Additionally, the proband presented with multiple skeletal abnormalities characteristic of MFS. Her height was 1.69 m (Figure 2A), with an arm span of 1.79 meters (Figure 2B), resulting in a span-to-height ratio of 1.06 (>1.05). Meanwhile, the proband exhibited pes planus (flat feet) (Figure 2C,D) and positive wrist signs (Figures 2E–G). CT imaging confirmed the pectus carinatum deformity, with a thoracic flatness index (TFI) of 3.95 (Figure 2H). x-ray imaging provided a definitive diagnosis of scoliosis (Figures 2I,J).

**Figure 3:**
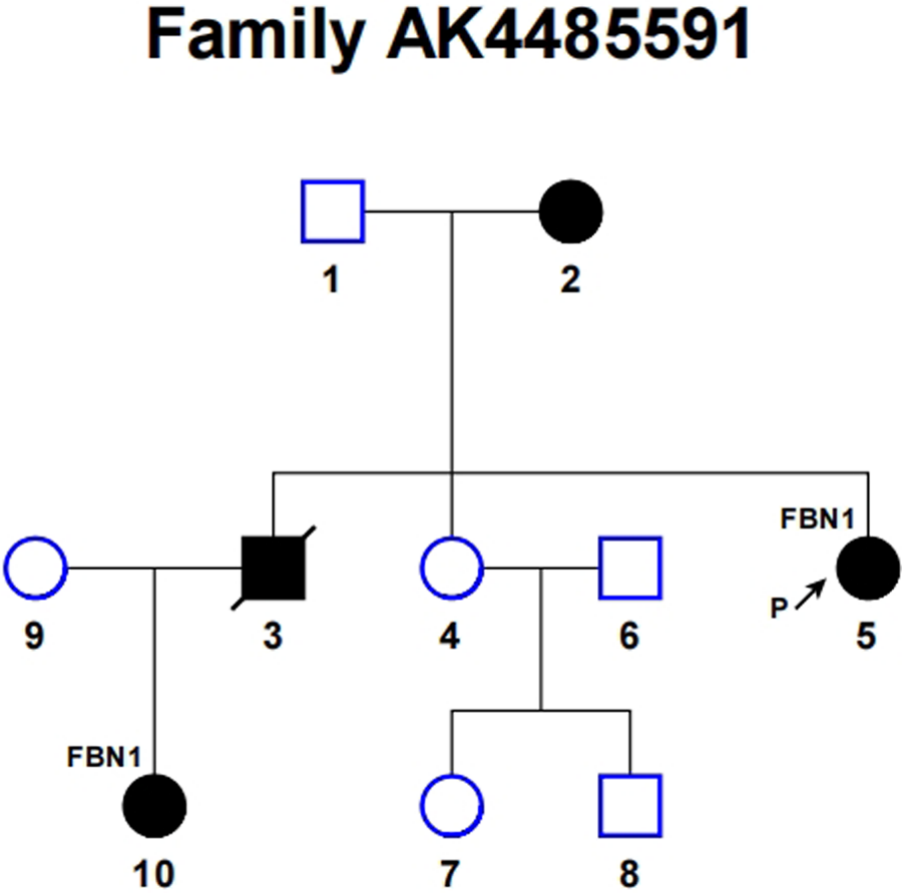
Pedigree of the family with Marfan syndrome. Solid symbols indicate affected patients, open symbols indicate unaffected subjects. The arrow points to the proband and slashed symbols denote deceased individuals. Squares represent males, and circles represent females.

Further investigation of the proband's family history revealed four affected individuals among ten family members, as schematized in Figure 3, consistent with an autosomal dominant inheritance pattern. The clinical manifestations of these four affected individuals are recorded in Supplementary Table 1. The patient underwent valve-sparing aortic root replacement (David Procedure) and total aortic arch replacement using a tetrafurcated graft with implantation of a specially designed frozen elephant trunk in the descending aorta (Sun's Procedure). Postoperatively, the patient underwent biweekly clinical follow-ups for three months. No treatment-related adverse events were reported during the monitoring period.

**Figure 4:**
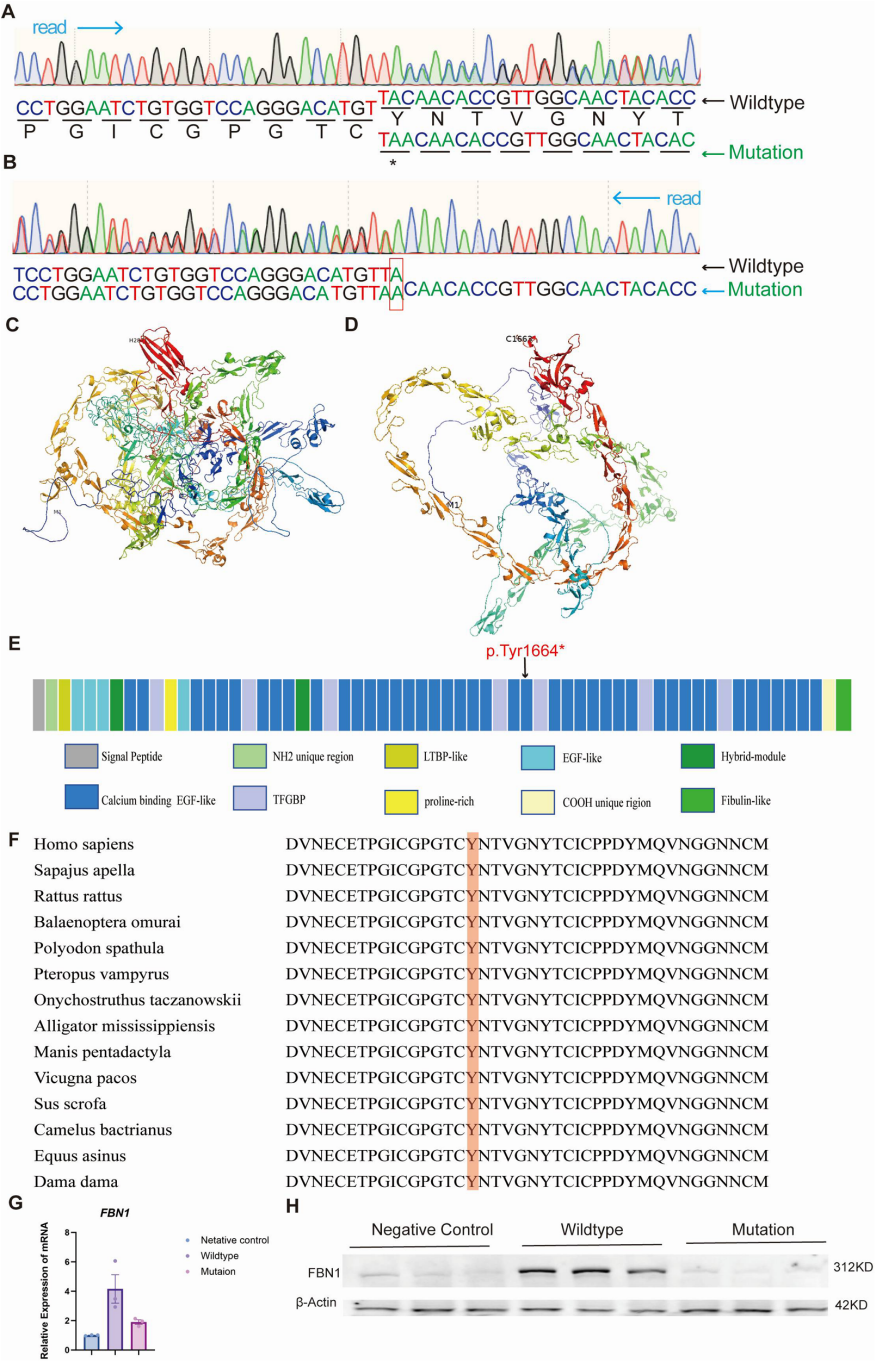
Identification of the *FBN1* mutation in the proband. **(A)** and **(B)** show the 5’ and 3’ end Sanger sequencing results, respectively. **(C)** and **(D)** illustrate the normal protein structure and the mutated protein model. **(E)** The *FBN1* gene has various functional regions, with the p.Tyr1664X mutation occurring in the calcium-binding EGF-like domain 24. **(F)** Orthologous protein sequence alignment of *FBN1* from different species, highlighting the conservation of tyrosine at codon 1664 in red. (**G,H**) HEK293A cells were transfected with an empty vector (negative control), a plasmid encoding wild-type FBN1, or a plasmid encoding mutant FBN1 (p.Tyr1664X). FBN1 mRNA and protein levels were determined by qRT-PCR (**G**) and Western blotting (**H**).

Whole-exome sequencing (WES) analysis of proband's peripheral blood DNA identified a novel frameshift mutation, c.4991dupA, in the *FBN1* gene. As a result, a truncated protein (p.Tyr1664X) was produced. This mutation was subsequently confirmed through Sanger sequencing (Figures 4A–B). AlphaFold2 was used to illustrate the potential impact of this mutation on the fibrillin-1 protein structure (Figures 4C,D). Multiple sequence alignments of the human fibrillin-1 protein revealed that this novel mutation occurred within a highly conserved region across different species (Figure 4F). We constructed both wild-type and c.4991dupA mutant plasmids of the *FBN1* gene. Following transfection into HEK293A cells, RNA and protein were extracted for quantitative reverse transcription PCR (qRT-PCR, Figure 4G) and Western blot analysis (Figure 4H). The results demonstrated that the frameshift mutation led to a premature termination codon, resulting in ​nonsense-mediated mRNA decay (NMD) with significantly reduced mRNA levels and ​the absence of detectable protein products on immunoblotting.

## 3 Materials and methods

### 3.1 Patients and clinical data

This study was conducted at The University of Hong Kong - Shenzhen Hospital and approved by its Medical Ethics Committee. Informed consent was obtained from all participants. We collected comprehensive family, medical, and personal histories from each participant. All subjects underwent thorough physical, ophthalmologic, radiological, and cardiovascular examinations. MFS diagnoses were established based on the revised Ghent criteria.

### 3.2 Whole exome sequencing and mutation identification

Whole exome sequencing (WES) and initial data analysis were performed by BGI Co, Ltd (Shenzhen, China). Genomic DNA was isolated from peripheral blood leukocytes using the QIAamp DNA Blood Kit (QIAGEN, Hilden, Germany) following the manufacturer's protocol.

To confirm the results via Sanger sequencing, polymerase chain reaction (PCR) amplification was performed using 2× Hieff PCR Master Mix (Yeasen Biotechnology; Cat. No. 10102ES03) under the following conditions: 95°C for 5 min (initial denaturation); 35 cycles of 98°C for 30 s, 60°C for 1 min, and 72°C for 1 min; and a final extension at 72°C for 5 min. Primer sequences are provided in Supplementary Table 2.

### 3.3 Bioinformatics analysis

We used AlphaFold2 (developed by DeepMind) for protein structure prediction. First, we obtained the target protein sequences from the UniProt database. Then, we conducted structure prediction using the latest version of AlphaFold2 with default model parameters (8).

Comparative amino acid sequence analysis of the human FBN1 protein was performed across different species using HomoloGene (https://www.ncbi.nlm.nih.gov/homologene/?term=FBN1). The potentially damaging effects of the mutation on the structure and function of FBN1 were predicted using PROVEAN (http://provean.jcvi.org/index) and MutationTaster (https://www.mutationtaster.org/).

### 3.4 Plasmid

Wild-type (WT) FBN1 plasmid (pLV3-CMV-FBN1-3×HA-CopGFP-Puro) was commercially purchased from Yanming Biotechnology (cat. no. YM00643). The mutant FBN1 plasmid was generated by site-directed mutagenesis via PCR amplification of the WT plasmid. Mutation sites were introduced into the primers validated by ​agarose gel electrophoresis and sanger sequence (prime sequence and sanger details provided in Supplementary Table 2).

### 3.5 Cell culture and transfection

​HEK293A was purchased from Procell (CL-0003, Wuhan, China) and maintained in DMEM supplemented with 10% FBS and 1% penicillin-streptomycin at 37°C with 5% CO₂. Cells were seeded in 6-well plates (2 × 10⁵ cells/well) 24 h prior to transfection. For each well, 2 µg of plasmid DNA (WT or mutant FBN1) was mixed with 4 µl jetPRIME transfection reagent (Polyplus, cat. no. 101000046) in jetPRIME® buffer, incubated for 15 min, and added dropwise to cells. The medium was changed 6 h post-transfection. Cells were harvested 48 h post-transfection for downstream assays.

### 3.6 RNA isolation and qPCR

Total RNA was extracted from transfected HEK293A cells using a cell RNA extraction kit (Magen, cat. no. MD021) according to the manufacturer's protocol. RNA concentration/purity was measured via Nanodrop. To eliminate potential plasmid and genomic DNA contamination, RNA samples were treated with DNase I using Vazyme DNA Wipe (Vazyme Biotech, cat. no. R323-01) according to the manufacturer's protocol. First-strand cDNA was synthesized from 1 µg of total RNA using HyperScript RT SuperMix for qPCR (APExBIO, cat. no. K1074-100) in a 20 µl reaction. The reaction was incubated at 42°C for 15 min for reverse transcription, followed by enzyme inactivation at 95°C for 1 min.

Primers targeting FBN1 and 18S genes were used (sequences provided in Supplementary Table 2). Reactions were performed on a Roche LightCycler 480 (Roche) with SGExcel FastSYBR Master (Sangon biotech, cat. no. B532955-0005). Cycling conditions: 95°C for 10 min, 40 cycles of 95°C for 20 s, 60°C for 20 s, 72°C for 20 s Data were analyzed using the ΔΔCt method, normalized to 18S.

### 3.7 Protein extraction and western blot analysis

Cells were lysed in RIPA buffer (Beyotime, cat. no. P0013B) containing protease inhibitors (Beyotime, cat. no. P1005). Lysates were centrifuged (12,000 × g, 15 min, 4°C), and supernatant protein concentration was quantified via BCA assay (Vazyme, cat. no. E112 -02). 20 µg protein was separated on 7.5% SDS-PAGE gels at 120 mV for 1 h and transferred to PVDF membranes at 90 mV for 3 h. Membranes were blocked with 5% non-fat milk in TBST for 1 h, then incubated with primary antibodies: anti-FBN1 (Abclonal, cat. no. A16677; 1:1000) and anti-β-actin (Proteintech, cat. no. 20536-1-AP; 1:1000) overnight at 4°C. HRP-conjugated secondary antibodies (1:5000, Cell Signaling) were applied for 1 h at RT. After washing out the unbound primary antibody with 1 × TBST buffer 3 times, the membranes were then incubated with HRP-conjugated anti-rabbit secondary antibody (C31460100, Invitrogen) at room temperature for 45 min. Immunoreactive bands were visualized by enhanced chemiluminescence solution, and ImageJ software was used for band intensity analysis.

## 4 Discussion

Marfan syndrome (MFS) is a severe multiorgan connective tissue disorder characterized by arachnodactyly, ectopia lentis, and thoracic aortic aneurysm and dissection (9). The most life-threatening manifestations of MFS are cardiovascular complications, particularly aortic root aneurysm and aortic dissection (10). The diagnostic criterion for MFS was most recently revised in 2010 and termed Ghent II nosology (11). This updated criterion emphasizes the assessment of systemic features, thoracic aortic status, and *FBN1* genetic testing for diagnosis (12).

The proband was admitted to our hospital due to type-A aortic dissection and was ultimately diagnosed with MFS following a series of examinations. Genetic testing confirmed the presence of an *FBN1* pathogenic variant, which can help to distinguish MFS from other heritable thoracic aortic disease syndromes. Because patients with MFS are at a much higher risk of developing life-threatening acute aortic dissection, early and accurate diagnosis is crucial for timely prevention and intervention.

In this study, we identified a novel nonsense mutation (c.4991dupA; p.Tyr1664X) in exon 40 of the *FBN1* gene in a Chinese family with Marfan syndrome. This mutation is a frameshift insertion in exon 40 of FBN1, introducing a premature termination codon (p.Tyr1664X) that truncates the fibrillin-1 protein. This mutation is absent from both UMD and ClinVar databases. Furthermore, this mutational mechanism differs from the predominant *FBN1* mutation types reported in the UMD database (*n* = 3,077 variants): Missense variants (1,815/3,077, 59%) and small deletions (331/3,077, 11%) collectively account for ∼70% of FBN1 mutations. Additionally, This mutation co-segregated with the MFS phenotype in the family and was absent in unaffected family members. Such truncating mutations in *FBN1* have previously been associated with more severe MFS phenotypes, particularly concerning aortic involvement (3, 13). This is consistent with our proband's presentation, which included significant aortic root dilation and dissection at a young age.

This location of this mutation encodes the cbEGF 24 domains, which are crucial for protein-protein interactions and calcium binding in fibrillin-1 (14). Our structural modeling using AlphaFold2 suggests that the truncation caused by this mutation will lead to significant alterations in the protein's tertiary structure. The loss of downstream cbEGF 24 domains is likely to affect the protein's ability to form proper microfibrils and interact with other components of the extracellular matrix. This structural disruption may explain the severe cardiovascular phenotype observed in our proband.

To assess the molecular consequences of the FBN1 c.4991dupA frameshift variant, wild-type and mutant constructs were transfected into HEK293A cells. qRT-PCR analysis revealed a significant reduction in FBN1 mRNA levels, indicative of nonsense-mediated mRNA decay (NMD) triggered by the premature termination codon (p.Tyr1664X). Western blot analysis confirmed complete loss of fibrillin-1 protein in mutant-expressing cells compared to wild-type controls, suggesting either NMD-dependent transcript elimination or proteasomal degradation of truncated protein.

The c.4991dupA (p.Tyr1664X frameshift variant in FBN1 was classified as pathogenic according to ACMG/AMP guidelines. This duplication generates a premature termination codon (PVS1: pathogenic very strong evidence), resulting in complete loss of fibrillin-1 protein expression in HEK293 cells (PS3: functional evidence). According to the ACMG Standards and Guidelines, this mutation is pathogenic. The molecular mechanism underlying MFS pathogenesis primarily involves the dysregulation of TGF-β signaling (15). *FBN1* mutations can lead to the formation of abnormal microfibrils, impairing their ability to sequester TGF-β (16). This results in elevated plasma TGF-β levels, leading to the overactivation of the TGF-β signaling pathway (8). Consequently, *FBN1* mutations enhance collagen production, reduce aortic compliance, and upregulate elastase and matrix metalloproteinases, contributing to the pathogenesis of MFS (17).

From a clinical perspective, the identification of this mutation has important implications for patient management and genetic counseling. The association of truncating mutations with more severe cardiovascular manifestations suggests that patients carrying this mutation may benefit from more frequent cardiovascular monitoring and potentially earlier intervention. Whole-exome sequencing (WES) can be prioritized for early detection of FBN1 mutations in high-risk families. Therapeutic agents such as angiotensin receptor blockers (ARBs, e.g., losartan) or β blockers may be initiated to prevent/slow aortic growth if predefined thresholds are met. Emerging approaches like CRISPR-Cas9-mediated gene correction or adeno-associated virus (AAV)-based FBN1 replacement hold promise for restoring functional fibrillin-1. While these strategies require further preclinical validation, they represent a transformative avenue for managing aortic aneurysms in genetically defined subsets of MFS. Collectively, this information can guide family planning decisions for affected individuals, ​as well as facilitate genetic screening, monitoring, timely pharmacological interventions, and future gene-targeted therapies.

In conclusion, we identified a novel nonsense mutation, c.4991dupA (p.Tyr1664X), in exon 40 of the *FBN1* gene in a Chinese family with Marfan syndrome. This mutation is predicted to result in a truncated fibrillin-1 protein and is associated with a severe cardiovascular phenotype. Our findings expand the known mutational spectrum of *FBN1* and enhance understanding of genotype-phenotype correlations in MFS. Furthermore, this discovery provides valuable insights into the molecular mechanisms underlying MFS, paving the way for improved early diagnosis and intervention.

## Data Availability

The wild and mutant FBN1 sequences presented in the study are publicly available. This data can be found here: Figshare (DOI: 10.6084/m9.figshare.28741658).
