# Supplementary material for: Case Report: A FBN1 frameshift-and-nonsense mutation and aortic dissection in Marfan syndrome
Source: Front Cardiovasc Med. 2025 Apr 23;12:1533138. doi: 10.3389/fcvm.2025.1533138 (PMC12055527; doi:10.3389/fcvm.2025.1533138)
Supplement: Supplementary file 1 [file Datasheet1.pdf]

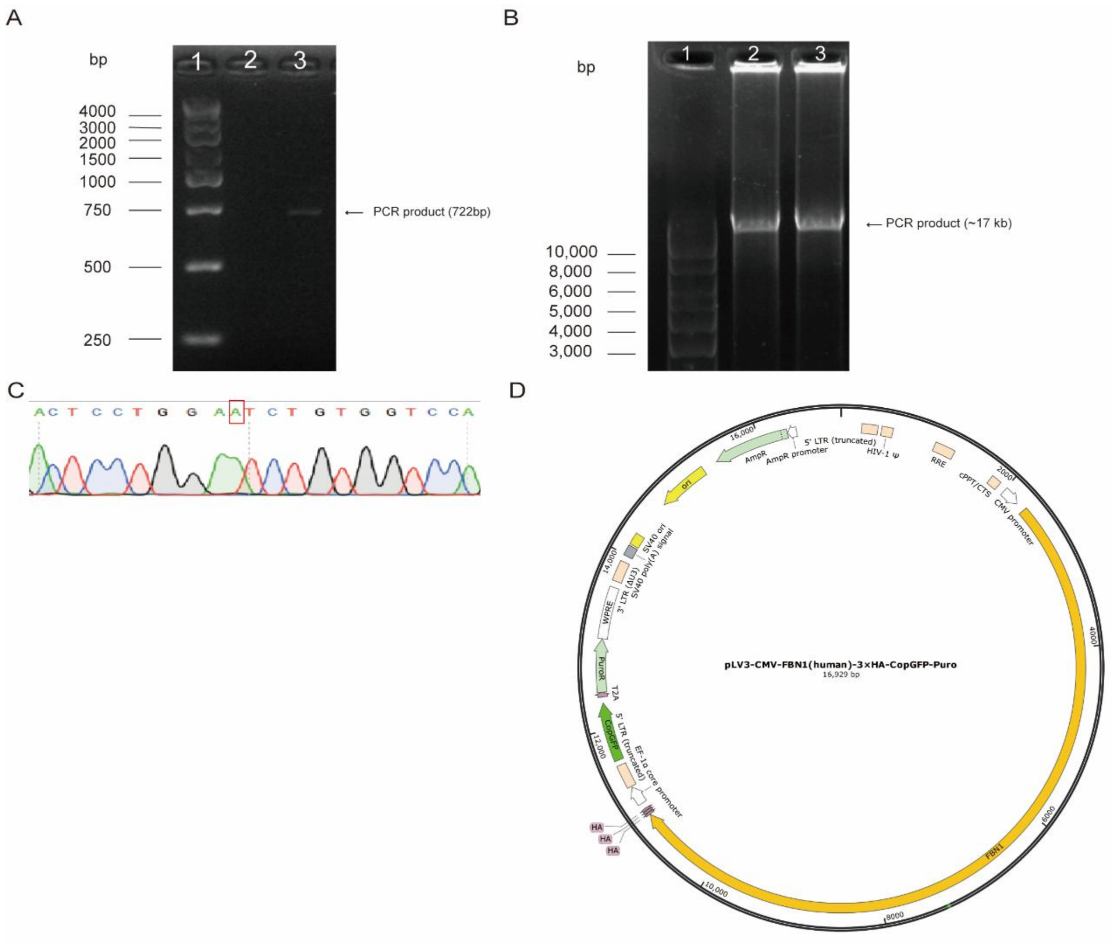

Supplemental Figure 1: Molecular validation of FBN1 variant constructs. (A) Agarose gel electrophoresis of proband’s genomic DNA extraction and PCR amplification. Lane 1: DNA ladder; Lane 2: Negative control (no-template PCR); Lane 3: PCR product (expected size: 722 bp). (B) Agarose gel electrophoresis of wild-type and mutant FBN1 plasmids. Lane 1: DNA ladder; Lane 2: Wild-type FBN1 plasmid; Lane 3: Mutant plasmid generated by site-directed mutagenesis. (C) Sanger sequencing chromatogram of the mutant plasmid. The red box highlights the duplicated adenine nucleotide (c.4991dupA) creating a premature stop codon. (D) Schematic representation of FBN1 plasmid structure. Critical elements are annotated: CMV promoter (white), FBN1 ORF (orange),3X HA tag (red), and ampicillin resistance gene (Light green).

Supplemental Table 1: Clinical details of the family members.

Table 1.	Clinical detail of the family members			
Basic information				
Family members	2	3	5	10
Age (years)	59	34	29	13
Gender	F	M	F	F
Weight (kg)	NA	65	46	47
Ocular System				
Ectopia lentis (lens dislocation)	-	-	-	-
Myopia	-	-	+	-
Abnormally flattened cornea	-	-	-	-
Early-onset cataractogenesis	-	-	-	-
Strabismus	-	-	-	-
Glaucoma	-	-	-	-
Retinal detachment	-	-	-	-
Cardiovascular System				
Aortic aneurysm and dissection	+	+	+	-
Mitral valve prolapse	-	-	-	-
Musculoskeletal System				
Arm span-to-height ratio >1.05	+	+	+	+
Wrist sign and Thumb sign	-	-	+	+
Pectus carinatum	-	-	+	-
Scoliosis	-	-	+	-
Arachnodactyly	-	-	+	+
pes planus	-	-	+	-
M, male; F, female;NA, not available				

Supplemental Table 2: Primer sequence

**Gene**	**Strand**	**Sequence 5'-3'**
Patient FBN1 mutant sequence prime	Forward	TCAGCCTCCCGAGTAGAGTA
	Reverse	GAAAACTCCCAACCACCCAC
FBN1 site-directed mutagenesis prime	Forward	GTCCAGGGACATGTTAACAACACCGTTGGCAACTACA
	Reverse	TAACATGTCCCTGGACCACAGATTCCA
18S	Forward	CGGCGACGACCCATTCGAAC
	Reverse	GAATCGAACCCTGATTCCCCGTC
FBN1 qRT-PCR prime	Forward	TGGACGAGTGTGAGGGTAAC
	Reverse	CGGGACACATGCACTTGTAG
FBN1 plasmid sanger sequence prime	Forward	CCGACCAAATCCTATCACCG
